# Equity in Health Care Access, Utilization, and Experiences for Latino Children in California by Parental Citizenship and Household Language, 2021–22

**DOI:** 10.1016/j.acap.2025.102856

**Published:** 2025-05-22

**Authors:** Clara B. Barajas, Dylan H. Roby, Jim P. Stimpson, Ninez A. Ponce, Gabriela E. Lazalde, Maria-Elena De Trinidad Young, Kathryn Kietzman, Arturo Vargas Bustamante, Alexandra C. Rivera-González, Brent A. Langellier, Jan M. Eberth, Mark Stehr, Alexander N. Ortega

**Affiliations:** Department of Health Management and Policy (CB Barajas, BA Langellier, JM Eberth, and M Stehr), Dornsife School of Public Health, Drexel University, Philadelphia, Pa; Center for Health Policy Research (DH Roby, NA Ponce, GE Lazalde, M-E De Trinidad Young, K Kietzman, AV Bustamante, and AN Ortega), Fielding School of Public Health, University of California, Los Angeles; Department of Health, Society, and Behavior (DH Roby), Program in Public Health, University of California Irvine; Peter O’Donnell School of Public Health (JP Stimpson), University of Texas Southwestern Medical Center, Dallas, Tex; Department of Health Policy and Management (NA Ponce and AV Bustamante), Fielding School of Public Health, University of California, Los Angeles; Department of Public Health (M-E De Trinidad Young and AC Rivera-González), School of Social Sciences, Humanities, and Arts, University of California, Merced; Thompson School of Social Work & Public Health (AN Ortega), University of Hawai’i at Mānoa, Honolulu, Hawaii

**Keywords:** children, health care quality, health care utilization, Hispanic, immigrants, Latino

## Abstract

**Objective:**

This study examines health care access, utilization, and experiences among Latino children in California by parental citizenship and household language.

**Methods:**

Merged data from the 2021–22 California Health Interview Survey and the follow-up Latino Youth Health Study of the same years were analyzed. Primary outcomes were parental reports of children’s health care access, utilization, and experiences in the past year. The main predictors were variables stratified by parental citizenship status (both citizen parents vs 1 citizen and 1 noncitizen parent or both noncitizen parents) and household language (English-only vs English-and-Spanish, or Spanish-only). Multivariable analyses were adjusted for parental education, family income, parent-reported child’s health status, child’s age, and child’s insurance.

**Results:**

Findings showed no significant differences in health care access across groups. However, children with both noncitizen parents and from Spanish-only households were more likely to have had well-child visits and general doctor visits than children with both citizen parents and in English-only households. Additionally, parents of children with both noncitizen parents were more likely to feel respected by doctors than those with both citizen parents. Conversely, compared to parents with both citizen parents, parents of children with 1 citizen and 1 noncitizen parent were less likely to report that doctors spent enough time with their children and less likely to express high satisfaction with their children’s health care.

**Conclusions:**

Patterns of health care access, utilization, and experiences among Latino children in immigrant families in California are improving, which are likely associated with recent inclusive health policies in the state.

Latino youth compose 25%, or 1 in 4, of all children in the United States,^1^ and nationally, they face persistent inequities in health care access and utilization compared to youth from other racial and ethnic groups.^2–4^ These inequities result from a complex interplay of factors spanning socioeconomic and cultural factors, family dynamics, community structures, exclusionary policies, and broader societal and political issues.^5^ These factors may also play a role in the health care experiences and perceived quality of care of Latino immigrant families, who are especially vulnerable to exclusionary health policies.

In California, 1 in 5 of all children live in mixed-status families (eg, families with members with different citizenship and legal authorization statuses),^6^ and 52% of all children are Latino.^1^ Exclusionary policies that limit eligibility for Medicaid programs make it difficult for mixed-status families to access health care.^7,8^ The limited availability of Spanish-speaking medical staff and interpreters also contributes to the difficulty of understanding and navigating the health care system, particularly for immigrant Spanish-speaking families.^9,10^

Over the past decade, California has enacted policies to improve access to health care. For instance, in May of 2016, the Health4All Kids program expanded full-scope Medi-Cal (California’s Medicaid program) coverage to all children under age 19, regardless of immigration status, as long as their families meet the income eligibility at or below 266% of the federal poverty level.^11,12^ The Health4All Kids program resulted in more than 250,000 children being enrolled in Medi-Cal.^13^ In addition to expanding Medi-Cal to all residents who meet the income eligibility threshold, California has taken steps to improve access to interpreter services for Medi-Cal beneficiaries with limited English proficiency.^14,15^

In 2023, Latino children in California had an uninsured rate of 3.8%,^16^ which is a notable decrease from the 4.5% uninsured rate in 2015.^17^ However, even with insurance coverage, studies have found that Latino children face disparities in health care utilization and quality of care.^4,18^ Given the implementation of inclusionary health policies targeted to immigrant families in California over the past 10 years, this study examines current patterns of health care access, utilization, and experiences for Latino children in the state by parental citizenship and household language.

## Methods

### Data Source

#### California Health Interview Survey

This study uses merged data from the 2021 and 2022 California Health Interview Surveys (CHIS) and the Latino Youth Health Study (LYHS). CHIS is the nation’s largest state population-representative health survey that collects annual data on health care access and utilization, demographics, health behaviors, and health outcomes, among other topics, from noninstitutionalized households in California.^19^ CHIS is weighted to represent the California population using a complex sampling methodology that considers various factors, including demographics, geographic location, and household characteristics.^20^

#### Latino Youth Health Study

LYHS is a CHIS follow-up survey to measure the multilevel factors that influence health care access and utilization for Latino youth and families. Immediately following the 2021–22 CHIS interviews, parents or guardians (ages 18 and older) of Latino youth (ages 0–17) in California who lived with at least one of their Latino youths and spoke either English or Spanish were invited to participate in LYHS. If they agreed, they were recontacted at a later date to complete the survey online or by telephone. Several methods were used to contact eligible participants, including mailed invitations and phone calls. Participants who completed the LYHS interview received a $60 gift card by mail or electronically.

The final LYHS sample consists of n = 1373 households. Of these, n = 939 families had data linked to 1 child (aged 0–11 years), n = 208 had data linked to 1 adolescent (aged 12–17), and n = 226 had data linked to 1 child and 1 adolescent. The LYHS sample is weighted to represent all parents in households with Latino youth and all Latino youth living with at least 1 parent. The survey weights for LYHS were created based on the 2021–22 eligible respondents who participated in the base CHIS interview. The adjusted base weights from the production CHIS^20^ were used for the base weights in LYHS.

For the current study, the final sample size is restricted to all adults ages 18 to 64 with Latino children ages 0 to 11 and who reported speaking English-only, Spanish-only, or English and Spanish at home (n = 1109). Families with only Latino adolescents (ages 12–17, n = 208) were excluded from this study because data on the citizenship statuses of both parents were collected only for households with a child under age 12. Families who spoke languages other than English-only, Spanish-only, or English and Spanish at home were excluded to minimize potential bias from language-related differences in health care experiences (n = 48). Finally, families with parents older than 64 years (n = 12, including 4 already excluded for language criteria) were excluded to reduce bias related to age, as this group may have different health care options, such as Medicare.

### Measures

#### Dependent Variables

The primary outcomes of interest were parent-reported measures of their child’s access to, use of, and experiences with health care in the past year. Four binary measures of access to health care included whether the child 1) had a usual source of care other than the emergency department (ED), 2) delayed or did not receive needed medical care, 3) delayed or did not receive needed prescription medicine, and 4) had to forgo necessary care (all coded as yes or no). In addition, 3 binary measures of health care utilization included whether the child had at least 1) 1 well-child visit (eg, physical or general-purpose check-up), 2) 1 doctor visit (eg, seeing any kind of medical doctor), or 3) 1 ED visit, all coded as yes or no.

There were 6 measures of parent-reported health care experiences in the past year. Five of the health care experiences focused on the communication interaction between the parent and the child’s health care provider, specifically whether the doctor: 1) spent enough time with the child, 2) explained things about the child’s health in a way that the parent could understand, 3) listened carefully to what the parent had to say, 4) showed respect for what the parent had to say, and 5) showed sensitivity to the parent’s values. These health care experiences were coded as yes if the parent always or usually had the type of health care experience, or no if the parent sometimes or never received the type of health care experience for their child. The sixth measure of health care experience examined whether the parent was satisfied with their child’s health care. This measure was coded as yes if the parent was very or somewhat satisfied, or no if the parent was very or somewhat dissatisfied.

#### Stratified Variables

The parental citizenship variable was stratified into 3 mutually exclusive categories: 1) Latino children with both citizen parents (reference group), which included US-born or naturalized (n = 737), 2) Latino children with 1 citizen and 1 noncitizen parent (n = 231), and 3) Latino children with both noncitizen parents (n = 141). Across all groups, 130 parents and 16 children were determined to be undocumented immigrants. In addition, these categories included single-parent (n = 376) and 2-parent households (n = 733). Similarly, the household language variable was stratified into 3 mutually exclusive categories: 1) Latino children living in households where only English is spoken (n = 367, reference group), 2) English and Spanish (n = 597), or 3) Spanish only (n = 145).

#### Covariates

Covariates included the reporting parent’s level of education (≤high school or > high school), family income measured as a percentage of the federal poverty level (0%–99%, 100%–199%, and ≥200%), parent-reported health status of the child (fair/poor, good, and very good/excellent), child’s age in years (0–3, 4–7, and 8–11), and child’s insurance status and type (private, public, or un-insured).

### Statistical Analyses

Analyses were performed using Stata MP 18.0^21^ and accounted for the complex survey sampling design, including sampling weights and strata, using the Taylor linearization method. First, analyses were performed to provide sample descriptive statistics. Statistically significant differences between the child groups were determined using Pearson chi-square tests with a threshold of *P* < 0.05. Second, separate logistic regression models were run to generate estimates for each of the dependent variables (ie, measures of health care access, utilization, and experiences) by the 2 stratified variables (ie, parental citizenship status and household language); children with both citizen parents and children in English-only households were the reference categories.

All logistic regression models were adjusted for the covariates described above. Stata’s margins command was used to obtain predicted probabilities for each of the outcomes and contrasts of the predicted margins across the groups of children. The design-adjusted Rao-Scott chi-square test was used to determine statistically significant differences between groups. A *P*-value less than 0.05 was used to determine statistical significance.

## Results

### Sample Characteristics

Descriptive characteristics of the sample by parental citizenship are presented in Table 1. The sample included children with both citizen parents (55.4%), 1 citizen and 1 noncitizen parent (21.4%), and children with both noncitizen parents (23.2%). No significant differences were observed in health care access or utilization across groups. However, children with 1 citizen and 1 noncitizen parent had a lower proportion of parents reporting that doctors always or usually spent enough time with their child (74.4%) compared to children with both citizen parents (87.1%) or both noncitizen parents (86.5%). Also, fewer parents in households with 1 citizen and 1 noncitizen parent reported being very or somewhat satisfied with their child’s health care (88.2%), compared to parents in households with 2 citizen parents (96.0%) or 2 noncitizen parents (93.9%); nonetheless, satisfaction remained high across all groups.

Additionally, households with both noncitizen parents had a higher proportion with a high school education or less and lower family income compared to the other groups. These households also had fewer children in very good or excellent health (78.0%) compared to those with both citizen parents (98.8%) or 1 citizen and 1 noncitizen parent (83.2%). A greater proportion of children with both noncitizen parents were older (8–11 years, 51.6%) and uninsured (7.0%) than their counterparts in other groups.

Descriptive characteristics by household language are presented in Table 2. Most households (53.7%) spoke both English and Spanish at home, while 24.8% spoke only English and 21.5% spoke only Spanish. No significant differences in health care access were found across the groups. However, significant differences emerged in health care utilization: a higher proportion of children in Spanish-only households had a well-child visit (95.1%) compared to those in English-only (83.8%) and English-and-Spanish households (89.3%). Although overall parental satisfaction with children’s health care was high, fewer parents in English-and-Spanish households reported being very or somewhat satisfied (91.3%) compared to parents in English-only (97.0%) and Spanish-only households (97.5%).

In addition, fewer parents in Spanish-only households had greater than a high school education (16%) compared to 60% in English-only households and 48.4% in English and Spanish households. Spanish-only households also had the highest proportion of parents with incomes at or below 99% of the federal poverty level (37.8%). Additionally, 3.9% of children in Spanish-only households were uninsured, compared to 0.8% in English-only and 2.1% in English-and-Spanish households.

### Multivariable Analyses

Table 3 shows the predicted probabilities from the multivariable analyses of parent-reported access to, use of, and experiences with the health care of their child by parental citizenship adjusted for parent’s education level, family income, parent-reported health status of the child, child’s age, and child’s insurance status and type. No significant differences in health care access were observed across the groups. However, for health care utilization, the predicted probability of parents reporting a well-child visit was 94.6% for children with both noncitizen parents, which was 8.3% points higher than for children with both citizen parents (*P*-value: < 0.05). Similarly, the predicted probability of having at least 1 doctor visit in the past year was 95.9% for children with both noncitizen parents, 7.6% points higher than their counterparts (*P*-value: < 0.05).

Significant differences in predicted probabilities by parental citizenship were found for 3 health care experience measures. Among children with 1 citizen and 1 noncitizen parent, the predicted probability of parents reporting that doctors spent enough time with their children was 73.7%, which was 11.9% points lower than for children with both citizen parents (*P*-value < 0.05). In contrast, children with both noncitizen parents had a predicted probability of parental satisfaction with their children’s health care of 95.8%, 7.9% points higher than those with both citizen parents (*P*-value < 0.05).

Table 4 presents the predicted probabilities from the adjusted multivariable analyses of parent-reported access to, use of, and experiences with health care for their children by household language. The predicted probability of parents reporting that their children had well-child visits was 89.3% in English and Spanish-speaking households and 95.9% in Spanish-only households, and both were significantly higher than among English-only households by 7.8% points (*P*-value < 0.05) and 14.4% points (*P*-value < 0.01), respectively. Children in Spanish-only households also had a higher predicted probability of having at least 1 general doctor visit (96.5%), which was 12.1% points higher than that of children in English-only households (*P*-value < 0.01). In addition, the only significant difference in health care experiences was that parents in English and Spanish-speaking households had a lower predicted probability of being satisfied with their children’s health care (91.0%), which was 5.4% points lower than in English-only households (*P* < 0.05).

## Discussion

### Key Findings

This study provides updated reports on health care access, utilization, and experiences among Latino children in California, with a focus on parents’ citizenship statuses and household language. We found that health care access patterns were largely similar across groups, but notable differences were observed in utilization and experiences. Latino children of both noncitizen parents and those in Spanish-only speaking households were more likely to have had well-child and general physician visits in the past year compared to children with both citizen parents and those in English-only speaking households. In addition, compared to children of both citizen parents, parents in households where children had both noncitizen parents were more likely to report that the children’s doctor showed respect for what they had to say. In contrast, parents in households where children had 1 citizen and 1 noncitizen parent reported less favorable health care experiences than those with both citizen parents, being less likely to report that doctors spent enough time with their children and less likely to report high satisfaction with their children’s health care.

### Comparisons With Previous Research

In contrast to the findings of our study, a study using 2011–12 CHIS found that Latino children with noncitizen parents or Spanish-only speaking households reported fewer physician visits and poorer communication compared to Latino children with both citizen parents and in English-only speaking households.^22^ Another study using 2011–14 CHIS found that Mexican-origin children in California with both citizen parents and with 1 naturalized parent were twice as likely to see a physician than children with both undocumented parents.^23^ Similarly, a 1997 study based in Los Angeles, California, reported that US-born Latino children with immigrant parents were twice as likely to lack access to health care as those with both citizen parents.^24^

While our study focused only on Latino children in California, the findings suggest that inequities in health care access and most measures of health care experiences are narrowing among Latino children in the state when examined by parents’ citizenship statuses and household language. However, for some measures of health care use, there appears to be a reverse pattern from previous studies in which children with both noncitizen parents have higher reports of health care use than those with both citizen parents.

### California’s Health Policies And Programs

California’s rather inclusive health policies and programs likely contributed to the improved health care utilization and overall reduced inequities in health care access and experiences observed among Latino children in our study, particularly those with both noncitizen parents and in Spanish-only speaking households. Programs such as Healthy San Francisco and MyHealthLA provided coverage for undocumented immigrants even before Medi-Cal expansion.^25,26^ Legislation improving interpreter services may have also enhanced care for Spanish-speaking families (ie, Assembly Bill 635 in 2016 and Senate Bill 165 in 2019).^14,15^ Latino children may have also had improved access to health care through programs such as Federally Qualified Health Centers, health windows at Mexican consulates, and other safety net clinics.^27,28^ Federally Qualified Health Centers, in particular, tend to be mission-driven and community-centered and provide comprehensive, culturally sensitive health care services to underserved communities, regardless of insurance status and demographics.^28^

While the study results are encouraging, we only focused on Latino youth, and it is important to recognize that inequities may still exist between Latino youth and other racial and ethnic groups even in California. Continued efforts are necessary to reduce gaps and achieve health equity. Efforts should include increasing provider participation in Medi-Cal, improving outreach to immigrant families, and supporting organizations that work with immigrant communities through funding initiatives. Additionally, considering that Spanish-speaking immigrants are especially vulnerable to mis- and disin-formation,^29,30^ it is essential that organizations facilitate access to accurate information about available health services and resources. In addition, the current political climate, which is marked by intense federal immigration enforcement,^31–33^ may further heighten fears and mistrust among immigrant families and discourage them from seeking care even for their children.^34–36^ To address these challenges, health organizations should actively foster a sense of safety and trust and ensure that immigrant families feel welcome and secure when accessing health care services.

### Limitations

First, we did not conduct pre-/post-policy analyses and thus cannot claim that the results observed are directly attributable to health policy changes. Second, CHIS uses a repeated cross-sectional design, and thus, we were unable to assess within-person changes over time. Third, with only Latino children included in LYHS, we were unable to compare patterns of health care access, utilization, and experiences with youth from other racial and ethnic groups. Nonetheless, our within-Latino group comparisons are very relevant given the consistent poor health care access and utilization patterns observed among Latino youth and the roles parental citizenship and household language use have played in inequities. Fourth, because of the relatively small sample size of undocumented immigrant parents in LYHS, we did not categorize the children by parental documentation status. Fifth, we did not examine the length of time parents have lived in the United States, which may have played a role in parent-reported health care experiences (eg, due to acculturation, awareness of the US health care system, or comparisons of health care experiences with those in their home countries).

## Conclusion

This study found a positive shift in health care access, use, and experience patterns for Latino children in California, particularly for those with noncitizen parents or in Spanish-only speaking households, which contrasts with earlier CHIS reports that observed inequities. The improvements may be associated, in part, with California’s inclusive health policies and programs implemented over the past decade, including expanded coverage for undocumented immigrants and enhanced interpreter services. Future research should examine whether immigrant parents’ positive reports of their children’s health care experiences reflect previously unmet health care needs in their home countries and also continue to monitor health care patterns among Latino children, with particular attention to the long-term effects of California’s policy initiatives. California’s approach to expanding health care access for immigrant families through policies like Medi-Cal expansion and targeted outreach may serve as a model for other states seeking to support immigrant communities and sustain access to care amid shifting federal policy priorities.

## Figures and Tables

**Table 1.** Characteristics of Latino children, ages 0–11, by parental citizenship

Variables	All Latino children		Child with both citizen parents		Child with one citizen parent		Child with both noncitizen parents		*P-value* *
	*n=1109*		*n=737*		*n=231*		*n=141*		
Health care access									
Had a usual source of care other than ED	1053	(94.7)	707	(94.8)	216	(94.8)	130	(94.7)	0.9988
Delayed/did not get needed medical care	82	(6.2)	49	(5.3)	22	(7.8)	11	(7.0)	0.6174
Delayed/did not get needed prescription medicine	41	(3.6)	26	(3.7)	12	(5.3)	3	(1.6)	0.2434
Had to forgo necessary care	37	(2.7)	20	(2.3)	11	(4.5)	6	(1.9)	0.2982
Health care utilization									
Had well-child visit	957	(89.2)	630	(87.9)	201	(89.2)	126	(92.3)	0.3939
Had ≥ 1 doctor visit	991	(91.2)	655	(90.1)	207	(91.2)	129	(93.7)	0.4925
Had ≥ 1 ED visit	145	(14.1)	97	(13.2)	30	(16.6)	18	(14.0)	0.7721
Health care experiences ^a^									
The doctor always/usually spent enough time with the child	844	(84.3)	567	(87.1)	166	(74.7)	111	(86.5)	**0.0388**
The doctor always/usually explained things about the child’s health in a way the parent could understand	931	(94.3)	623	(95.5)	189	(91.4)	119	(94.3)	0.3537
The doctor always/usually listened carefully to what the parent had to say	899	(89.9)	598	(91.1)	183	(87.4)	118	(89.5)	0.6650
The doctor always/usually showed respect for what the parent had to say	907	(89.7)	602	(91.0)	183	(82.3)	122	(93.1)	0.0681
The doctor always/usually showed sensitivity to values	878	(86.5)	591	(89.2)	176	(82.2)	111	(84.0)	0.2599
Parent was very/somewhat satisfied with the health care of their child	935	(94.1)	627	(96.1)	188	(88.2)	120	(93.9)	**0.0243**
Other measures									
Parent’s education level									**<0.001**
≤ High School	298	(55.4)	131	(37.8)	73	(63.7)	98	(89.6)	
> High School	811	(44.6)	606	(62.2)	158	(36.3)	47	(10.4)	
Federal poverty level									**<0.001**
0–99%	218	(25.1)	120	(21.1)	53	(26.1)	45	(33.8)	
100–199%	261	(27.9)	123	(18.8)	73	(31.4)	65	(46.4)	
≥ 200%	630	(47.0)	494	(60.1)	105	(42.5)	31	(19.8)	
Child’s general health status									**0.0383**
Fair/poor	35	(4.7)	16	(3.0)	11	(7.9)	8	(5.9)	
Good	93	(9.6)	49	(7.2)	23	(8.9)	21	(16.1)	
Very good/excellent	981	(85.7)	672	(98.8)	197	(83.2)	112	(78.0)	
Child’s age (years)									**0.0160**
0–3	321	(31.4)	228	(34.0)	65	(38.6)	28	(18.5)	
4–7	348	(30.7)	220	(32.4)	80	(27.2)	48	(29.9)	
8–11	65	(37.9)	289	(33.6)	86	(34.3)	65	(51.6)	
Child’s insurance status and type									**<0.001**
Private	567	(40.8)	468	(57.6)	73	(23.6)	26	(16.5)	
Public	523	(57.0)	265	(42.1)	152	(74.6)	106	(76.5)	
Uninsured	19	(2.2)	4	(0.3)	6	(1.8)	9	(7.0)	
Languages spoken at home									**<0.001**
English only	367	(24.8)	340	(41.2)	23	(8.2)	4	(1.1)	
English and Spanish	597	(53.7)	371	(53.4)	163	(60.7)	63	(50.7)	
Spanish only	145	(21.5)	26	(5.5)	45	(31.1)	74	(48.2)	

Source: Latino Youth Health Study and the California Health Interview Survey, 2021 and 2022 waves.

ED indicates emergency department.

The sample was limited to Latino adults ages 18 to 64 with children ages 0 to 11 who reported speaking English only, English and Spanish, or Spanish only at home (n = 1109), including single-parent and 2-parent households.

Data are from the reporting parent, expressed as sample frequencies and weighted column percentages in parentheses, for the past 12 months unless otherwise indicated.

* Boldface indicates statistical significance.

† For health care experiences, the sample was limited to children who had seen a doctor in the past 12 months (n = 991).

**Table 2.** Characteristics of Latino Children, Ages 0 to 11, by Household Language

Variables	All Latino Children		English Only		English and Spanish		Spanish Only		*P*-value*
	n = 1109		n = 367		n = 597		n = 145		
Health care access									
Had a usual source of care other than ED	1053	(94.7)	354	(95.3)	567	(93.9)	132	(96.2)	0.5671
Delayed/did not get needed medical care	82	(6.2)	21	(4.7)	50	(7.9)	11	(4.0)	0.1877
Delayed/did not get needed prescription medicine	41	(3.6)	353	(3.4)	575	(4.3)	140	(2.0)	0.4082
Had to forgo necessary care	37	(2.7)	8	(2.1)	24	(3.4)	5	(1.5)	0.4559
Health care utilization									
Had well-child visit	957	(89.2)	300	(83.8)	519	(89.3)	138	(95.1)	**0.0277**
Had ≥1 doctor visit	991	(91.2)	320	(86.7)	532	(91.4)	139	(95.7)	0.0710
Had ≥1 ED visit	145	(14.1)	50	(15.8)	80	(14.6)	15	(10.8)	0.6318
Health care experiences^†^									
The doctor always/usually spent enough time with the child	844	(84.3)	278	(86.4)	443	(81.7)	123	(88.2)	0.3304
The doctor always/usually explained things about the child’s health in a way the parent could understand	931	(94.3)	308	(96.9)	494	(92.7)	129	(95.7)	0.1324
The doctor always/usually listened carefully to what the parent had to say	899	(89.9)	295	(90.9)	476	(87.9)	128	(93.7)	0.2147
The doctor always/usually showed respect for what the parent had to say	907	(89.7)	295	(91.9)	485	(88.4)	127	(90.4)	0.6373
The doctor always/usually showed sensitivity to values	878	(86.5)	290	(89.5)	466	(83.6)	122	(90.0)	0.2024
Parent was very/somewhat satisfied with the health care of their child	935	(94.1)	307	(97.0)	496	(91.3)	132	(97.5)	**0.0027**
Other measures									
Parent’s education level									< **0.001**
≤High school	298	(55.4)	63	(39.2)	146	(51.6)	89	(83.4)	
> High school	811	(44.6)	304	(60.8)	451	(48.4)	56	(16.6)	
Federal poverty level									< **0.001**
0%–99%	218	(25.1)	60	(23.3)	102	(20.8)	56	(37.8)	
100%–199%	261	(27.9)	56	(17.5)	154	(28.1)	53	(39.6)	
≥200%	630	(47.0)	102	(59.1)	341	(51.2)	36	(22.6)	
Child’s general health status									0.2333
Fair/poor	35	(4.7)	5	(3.7)	22	(4.9)	8	(5.5)	
Good	93	(9.6)	21	(5.3)	45	(9.4)	27	(15.1)	
Very good/excellent	981	(85.7)	341	(91.0)	530	(85.7)	110	(79.4)	
Child’s age (year)									0.3498
0–3	321	(31.4)	100	(38.1)	184	(30.6)	37	(25.6)	
4–7	348	(30.7)	108	(29.0)	193	(31.9)	47	(29.7)	
8–11	440	(37.9)	159	(33.0)	220	(37.5)	61	(44.7)	
Child’s insurance status and type									< **0.001**
Private	567	(40.0)	229	(57.1)	315	(44.4)	23	(12.9)	
Public	523	(57.0)	135	(42.1)	269	(53.5)	119	(83.2)	
Uninsured	19	(2.2)	3	(0.8)	13	(2.1)	3	(3.9)	
Parental citizenship									< **0.001**
Child with both citizen parents	737	(55.4)	340	(91.9)	371	(55.0)	26	(14.1)	
Child with 1 citizen parent	231	(21.4)	23	(7.1)	163	(24.2)	45	(31.0)	
Child with no citizen parents	141	(23.2)	4	(1.0)	74	(20.9)	63	(54.8)	

Source: Latino Youth Health Study and the California Health Interview Survey, 2021 and 2022 waves.

ED indicates emergency department.

The sample was limited to Latino adults ages 18 to 64 with children ages 0 to 11 who reported speaking English only, English and Spanish, or Spanish only at home (n = 1090), including single-parent and 2-parent households.

Data are from the reporting parent, expressed as sample frequencies and weighted column percentages in parentheses, for the past 12 months unless otherwise indicated.

* Boldface indicates statistical significance.

† For health care experiences, the sample was limited to children who had seen a doctor in the past 12 months (n = 991).

**Table 3.** Predicted Probabilities From Multivariable Analysis of Parent-Reported Health Care Access, Utilization, and Experiences of Latino Children, Ages 0 to 11, by Parental Citizenship

Variables	Child With Both Citizen Parents n = 737	Child With 1 Citizen Parent n = 231	Child With Noncitizen Parents n = 141
	ME	ME Difference (95% CI)	ME Difference (95% CI)
Health care access			
Has a usual source of care other than ED	94.17	94.95	95.67
	Reference	0.78 (−4.06, 5.64)	1.5 (−4.01, 7.01)
Delay/did not get needed medical care	5.0	7.38	8.64
	Reference	2.34 (−2.19, 6.88)	3.60 (−4.38, 11.60)
Delay/did not get needed prescription medicine	3.96	5.21	1.47
	Reference	1.24 (−3.20, 5.70)	−2.49 (−6.00, 1.01)
Forgone medical care	2.85	4.24	1.34
	Reference	1.38 (−2.68, 5.46)	−1.51 (−4.97, 1.94)
Health care utilization			
Had well-child visit	86.31	88.73	94.59
	Reference	2.41 (−4.38, 9.22)	**8.27 (1.7, 14.79)** *
Had ≥1 doctor visit	88.32	91.04	95.94
	Reference	2.72 (−3.57, 9.01)	**7.62 (1.68, 13.55)** *
Had ≥1 ED visit	14.08	14.20	13.99
	Reference	0.11 (−7.68, 7.92)	−0.09 (−8.75, 8.55)
Health care experiences^†^			
The doctor always/usually spent enough time with the child	85.63	73.73	89.88
	Reference	−11.90 (−22.94, −0.85)*	4.24 (−3.10, 11.59)
The doctor always/usually explained things about the child’s health in a way the parent could understand	94.17	91.37	96.49
	Reference	−2.80 (−8.91, 3.29)	2.31 (−1.81, 6.44)
The doctor always/usually listened carefully to what the parent had to say	89.72	86.70	92.34
	Reference	−3.01 (−11.15, 5.12)	2.62 (−4.22, 9.46)
The doctor always/usually showed respect for what the parent had to say	88.23	82.96	95.81
	Reference	−5.23 (−14.45, 3.93)	**7.58 (1.15, 14.00)** *
The doctor always/usually showed sensitivity to values	88.05	81.90	87.06
	Reference	−6.14 (−15.38, 3.08)	−0.99 (−9.82, 7.83)
Parent was very/somewhat satisfied with the health care of their child	96.01	88.10	95.06
	Reference	−7.90 (−14.99, −0.81)*	−0.94 (−5.89, 4.01)

Source: Latino Youth Health Study and the California Health Interview Survey, 2021 and 2022 waves.

ED indicates emergency department; ME, marginal effect.

The sample was limited to Latino adults ages 18–64 with children ages 0 to 11 who reported speaking English only, English and Spanish, or Spanish only at home (n = 1090), including single-parent and 2-parent households.

Health care access, utilization, and experiences are from the past 12 months unless otherwise indicated. Independent regression models were run for each of the dependent variables. All models were adjusted for parent education level, federal poverty level, parent-reported health status of the child, the child’s age, and the child’s insurance status and type. Percent differences may not be exact due to rounding.

* Boldface indicates statistical significance (P < 0.05).

† For health care experiences, the sample was limited to children who had seen a doctor in the past 12 months (n = 991).

**Table 4.** Predicted Probabilities From Multivariable Analysis of Parental-Reported Health Care Access, Utilization, and Experiences of Latino Children, Ages 0 to 11, by Household Language

Variables	English Only n = 364	English and Spanish n = 584	Spanish Only n = 142
	ME	ME Difference (95% CI)	ME Difference (95% CI)
Health care access			
Has a usual source of care other than ED	94.93	93.99	96.38
	Reference	−0.94 (−6.18, 4.29)	1.45 (−4.10, 7.06)
Delay/did not get needed medical care	4.85	7.51	4.35
	Reference	2.66 (−1.95, 7.27)	−0.50 (−6.04, 5.03)
Delay/did not get needed prescription medicine	3.4	4.37	1.90
	Reference	0.94 (−2.43, 4.32)	−1.52 (−4.90, 1.85)
Forgone medical care	2.64	3.25	1.34
	Reference	0.60 (−3.69, 4.91)	−1.29 (−5.98, 3.39)
Health care utilization			
Had well-child visit	81.51	89.32	95.90
	Reference	**7.80 (0.16, 15.44)** *	**14.39 (5.72, 23.05)** **
Had ≥1 doctor visit	84.33	91.46	96.53
	Reference	7.12 (−0.46, 14.72)	**12.19 (3.70, 20.69)** **
Had ≥1 ED visit	17.28	14.58	9.67
	Reference	−2.70 (−9.72, 4.31)	−7.61 (−16.91, 1.67)
Health care experiences^†^			
The doctor always/usually spent enough time with the child	85.07	81.34	89.76
	Reference	−3.72 (−11.41, 3.97)	4.69 (−4.87, 14.26)
The doctor always/usually explained things about the child’s health in a way the parent could understand	95.84	92.67	96.62
	Reference	−3.16 (−7.20, 0.87)	0.78 (−3.85, 5.41)
The doctor always/usually listened carefully to what the parent had to say	89.17	87.45	95.12
	Reference	−1.72 (−8.51, 5.07)	5.95 (−1.25, 13.15)
The doctor always/usually showed respect for what the parent had to say	89.60	87.86	92.96
	Reference	−1.74 (−8.88, 5.40)	3.35 (−4.79, 11.51)
The doctor always/usually showed sensitivity to values	87.18	83.14	92.22
	Reference	−4.03 (−11.76, 3.68)	5.04 (−3.05, 13.14)
Parent was very/somewhat satisfied with the health care of their child	96.43	90.98	98.03
	Reference	−5.44 (−9.91, −0.97)*	1.59 (−1.58, 4.77)

Source: Latino Youth Health Study and the California Health Interview Survey, 2021 and 2022 waves.

ED indicates emergency department; ME, marginal effect.

Boldface indicates statistical significance (*P < 0.05, **P < 0.01).

The sample was limited to Latino adults ages 18–64 with children ages 0–11 who reported speaking English only, English and Spanish, or Spanish only at home (n = 1090), including single-parent and 2-parent households. Health care access, utilization, and experiences are from the past 12 months unless otherwise indicated.

Independent regression models were run for each of the dependent variables. All models were adjusted for parent education level, federal poverty level, parent-reported health status of the child, the child’s age, and the child’s insurance status and type. Percent differences may not be exact due to rounding.

† For health care experiences, the sample was limited to children who had seen a doctor in the past 12 months (n = 991).

## WHAT’S NEW

In California, differences in Latino children’s health care access, utilization, and experiences by parental citizenship and household language appear to be narrowing, with notable improvements in utilization for children with noncitizen parents and those from Spanish-only speaking households.
